# Analysis of Structures, Functions, and Epitopes of Cysteine Protease from *Spirometra erinaceieuropaei* Spargana

**DOI:** 10.1155/2013/198250

**Published:** 2013-12-12

**Authors:** Li Na Liu, Jing Cui, Xi Zhang, Tong Wei, Peng Jiang, Zhong Quan Wang

**Affiliations:** Department of Parasitology, Medical College, Zhengzhou University, 40 Daxue Road, Zhengzhou 450052, China

## Abstract

*Spirometra erinaceieuropaei* cysteine protease (SeCP) in sparganum ES proteins recognized by early infection sera was identified by MALDI-TOF/TOF-MS. The aim of this study was to predict the structures and functions of SeCP protein by using the full length cDNA sequence of SeCP gene with online sites and software programs. The SeCP gene sequence was of 1 053 bp length with a 1011 bp biggest ORF encoding 336-amino acid protein with a complete cathepsin propeptide inhibitor domain and a peptidase C1A conserved domain. The predicted molecular weight and isoelectric point of SeCP were 37.87 kDa and 6.47, respectively. The SeCP has a signal peptide site and no transmembrane domain, located outside the membrane. The secondary structure of SeCP contained 8 **α**-helixes, 7 **β**-strands, and 20 coils. The SeCP had 15 potential antigenic epitopes and 19 HLA-I restricted epitopes. Based on the phylogenetic analysis of SeCP, *S. erinaceieuropaei* has the closest evolutionary status with *S. mansonoides*. SeCP was a kind of proteolytic enzyme with a variety of biological functions and its antigenic epitopes could provide important insights on the diagnostic antigens and target molecular of antisparganum drugs.

## 1. Introduction

Sparganosis is a serious parasitic zoonosis caused by infection with spargana, the plerocercoid larvae of some *Diphyllobothrium* tapeworms that belong to the genus *Spirometra* [1]. The most important species of the genus *Spirometra* tapeworms with plerocercoids that can produce sparganosis in human include *Spirometra erinaceieuropaei* (syn. *Spirometra erinacei* or *Spirometra mansoni*) which is the most common in Asia, and *Spirometra mansonoides* which is mainly distributed in North America [2]. The adults are intestinal parasites of some species of Canidae and Felidae; the first intermediate hosts are freshwater copepods (cyclops), whereas the second intermediate or paratenic hosts belong to different species of vertebrates (frogs, snakes, pigs, etc.) [3, 4]. Human is an accidental host. Human infection results mainly from drinking raw water contaminated with cyclops harboring procercoid, ingesting raw fleshes of frogs and snakes infected with plerocercoids, or placing frog or snake flesh on open wound for treatment of skin ulcers or eye inflammations [5, 6].

Human sparganosis is reported in many countries of the world but is most common in Eastern Asia and the Far East [7]. Sparganosis poses a serious threat to human health; the plerocercoids usually lodge in the subcutaneous tissues and muscles but sometimes invade the abdominal cavity, eye, and central nervous system causing blindness, seizures, headache, epilepsy, paralysis, and even death [8]. Ocular sparganosis is especially prevalent in China and Vietnam [9]. The clinical diagnosis of sparganosis is rather difficult and often misdiagnosed because the larvae have no predilection site in humans and the specific signs or symptoms are lacking. A definite diagnosis of subcutaneous sparganosis can be achieved by detection of the larvae in a biopsy specimen from the lesion, but the confirmative diagnosis is very difficult for visceral and cerebral sparganosis since the larva is found only by surgical removal [10]. The ELISA using the crude or excretory-secretory (ES) antiagens of plerocercoids has high sensitivity for the detection of sparganum infection in humans, but the main disadvantage is the false negative results during the early stage of infection and the cross-reactions with serum samples from patients with other parasitic diseases (cysticercosis, paragonimiasis, clonorchiasis, etc.) [11, 12].

In order to separate the early specific diagnostic antiagens, the ES proteins of *S. erinaceieuropaei* sparganum were analyzed by two-dimensional electrophoresis (2DE) and Western blot probed with early sera from infected mice at 14 days after infection. Three immunoreactive protein spots were successfully identified by MALDI-TOF/TOF-MS and characterized as the *S. erinaceieuropaei* cysteine protease (SeCP) [13]. In this paper, the full-length cDNA sequence of SeCP (GenBank accession no. 1834307) was analyzed; its structure and function were predicted by using bioinformatics techniques.

## 2. Materials and Methods

The full-length cDNA sequence of SeCP (GenBank accession no. 1834307) was used in this study. The structure domain and function domain were predicted by online analysis http://smart.embl-heidelberg.de/. The amino acid sequence was submitted to http://www.expasy.org/tools/protparam.html and its physical and chemical properties were predicted. Signal peptide was predicted by a web-based tool (http://www.cbs.dtu.dk/services/SignalP/), and subcellular localization was predicted using http://psort.nibb.ac.jp/form2.html. Hydrophilic prediction was predicted at http://www.expasy.org/cgi-bin/protscale.pl. Transmembrane domain was predicted through http://www.cbs.dtu.dk/services/TMHMM-2.0/. The secondary structures were constructed using the software PSIPRED v3.0 http://bioinf.cs.ucl.ac.uk/psipred/ [14, 15]. The 3D models of proteins were constructed by I-TASSER, a protein structure server on the website http://zhanglab.ccmb.med.umich.edu/I-TASSER/, which is considered to predict protein 3D structures that have more than 100 amino acids [16–18]. Visual molecular dynamics (VMD) was used to read standard Protein Data Bank (PDB) files and display the contained structure [19–21]. VMD is a molecular visualization software for displaying, animating, and analyzing large biomolecular systems using 3D graphics and built-in scripts http://www.ks.uiuc.edu/Research/vmd/. Amino acid sequence was submitted to http://www.cbs.dtu.dk/services/BepiPred/ in order to predict its antigen epitopes. Conserved HLA-restricted CD8+ T cells epitopes were also predicted using the software from IEDB http://www.immuneepitope.org/ which could identify novel HLA-class I restricted CD8^+^T cell epitopes.

Other cysteine protease amino sequences of model organisms of other parasites used in this study were obtained from GenBank (http://www.ncbi.nlm.nih.gov/Genbank/index.html) and listed as follows: *Clonorchis sinensis* (AAD29130.1), *Homo sapiens* (CAB42883.1), *Spirometra mansonoides* (AAB17051.1), *Taenia solium* (BAH03395.1), *Paragonimus westermani* (AAF21457.2), *Schistosoma japonicum* (CAX71578.10), *Schistosoma mansoni* (P25792.1), *Taenia pisiformis* (AEE69034.1), *Haemonchus contortus* (ACS36090.1), *Entamoeba histolytica* (CAA62836.1), *Trichinella spiralis* (XP_003377240.1), *Plasmodium vivax* (AAA60368.1), *Brugia malayi* (XP_001896823.1), *Echinococcus multilocularis* (BAF02516.1), *Arabidopsis thaliana* (AAB67626.1), *Mus musculus* (AAA37445.1), *Drosophila melanogaster* (AAB18345.1), *Caenorhabditis elegans* (AAA98785.1), *Haemaphysalis longicornis* (BAH86062.1), and *Aedes aegypti* (ABE72970.1). The multiple sequence alignment of SeCP and the above-mentioned sequences were carried out by Clustal X; then, molecular evolutionary tree was constructed by MEGA4.1 [22]. Phylogenies were estimated under the neighbor-joining (N-J) method [23].

## 3. Results

### 3.1. The Basic Properties of SeCP Sequence

**Figure 1:**
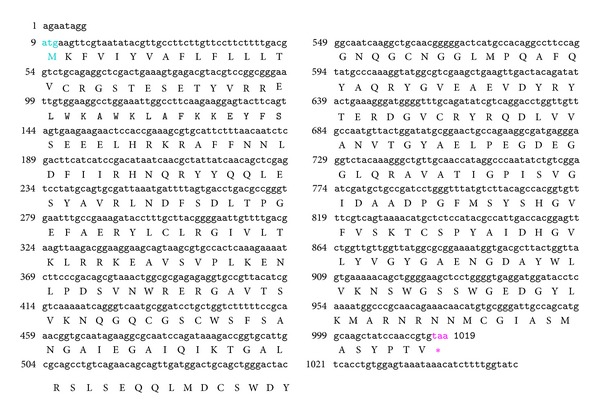
Sequences and amino acid residues of SeCP. The SeCP sequence was of 1 053 bp length with a 1011 bp biggest OFR from 9 bp (ATG) to 1019 bp (TAA), which encoded 336-amino acid protein with 3′UTR locating at the positions 1020–1053 bp.

The SeCP sequence was of 1 053 bp length with a 1011 bp biggest OFR from 9 bp (ATG) to 1019 bp (TAA), which encoded 336-amino acid protein with 3′UTR locating at the positions 1020–1053 bp. Nucleotide sequence and deduced amino acid sequence were shown in Figure 1.

### 3.2. Physical and Chemical Properties of SeCP

The SeCP had the molecular weight of 37.87 kDa and theoretical isoelectric point (pI) of 6.47. Extinction coefficients are 74300 M^−1^ cm^−1^, at 280 nm measured in water, assuming all pairs of Cys residues form cysteines. The half-life was 30 h, >20 h, and >10 h in mammalian reticulocytes (*in vitro*), yeast (*in vivo*), and *Escherichia coli* (*in vivo*), respectively. The instability index (II) was computed to be 32.11. This classifies the protein as stable. Aliphatic index is 75.74. Grand average of hydropathicity (GRAVY) is −0.321.

### 3.3. Structural Domain, Hydrophobicity, Signal Peptide, Subcellular Localization, and Transmembrane Domain

**Figure 2:**
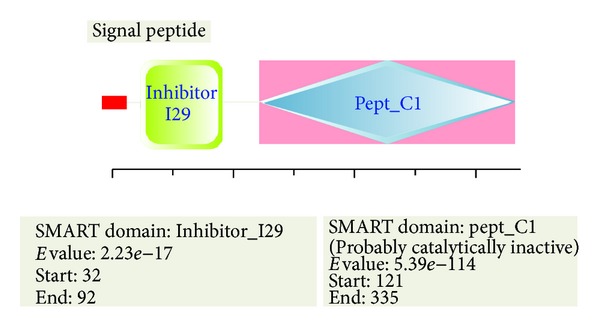
Prediction of structure domains of SeCP by SMART servers. The confidently predicted SeCP structure domains contained a complete cathepsin propeptide inhibitor domain (I29) located at 32aa–92aa and a peptidase_C1A with an active site located at 39aa–303aa and a S2 subsite 189aa–330aa, which has the function of cysteine-type peptidase activity.

**Figure 3:**
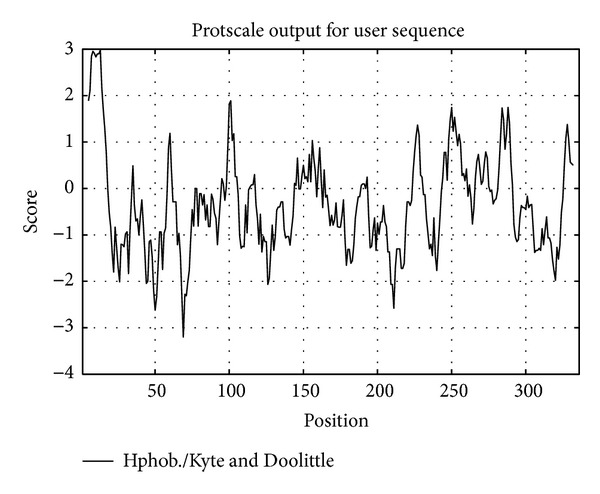
Hydrophobicity of SeCP. The SeCP protein has an obvious hydrophobic regions at 5′ predicted by using the scale Hphob./Kyte and Doolittle.

The confidently predicted SeCP structure domains contained a complete cathepsin propeptide inhibitor domain (I29) located at 32aa–92aa and a peptidase_C1A with an active site located at 39aa–303aa and a S2 subsite 189aa–330aa, which has the function of cysteine-type peptidase activity (Figure 2). Using the scale Hphob./Kyte and Doolittle, the SeCP protein has an obvious hydrophobic regions at 5′ (Figure 3).

**Figure 4:**
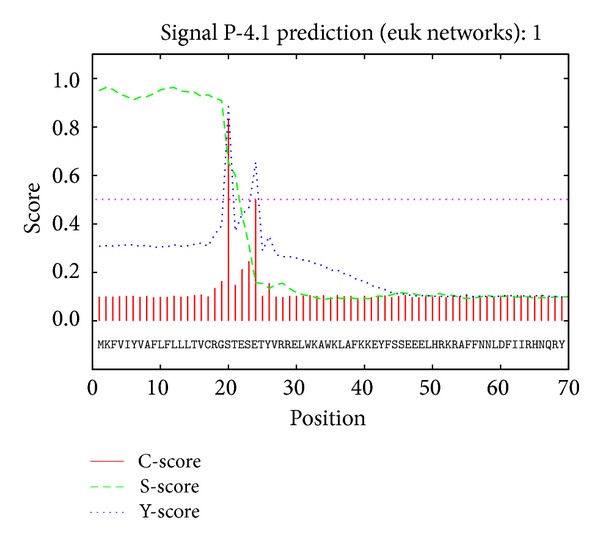
Prediction of SeCP signal peptide. There was a peak fraction at 19aa residue position and the score was 3.87 which was high enough with split site. The SeCP protein had a cleavable signal peptide (1 to 19) with possible cleavage site between 19aa and 20aa.

The prediction results of SeCP signal by Signal P-4.1 showed that there was a peak fraction at 19aa residue position (Figure 4). The score was 3.87 which was high enough with split site. So, the SeCP protein had a cleavable signal peptide (from 1 to 19) and with possible cleavage site between 19aa and 20aa.

Results of the k-NN prediction of SeCP suggested that the peptide chain was located in the extracellular (including cell wall), vacuolar, mitochondrial, and endoplasmic reticulum, with the possibility of 55.6%, 22.2%, 11.1%, and 11.1%, respectively. The maximum possible location was in the extracellular (*k* = 23).

Prediction of transmembrane domain of SeCP with TMHMM Server v. 2.0 suggested that the SeCP had no transmembrane domain, located outside the membrane.

### 3.4. 2D Structure Alignment for SeCP

**Figure 5:**
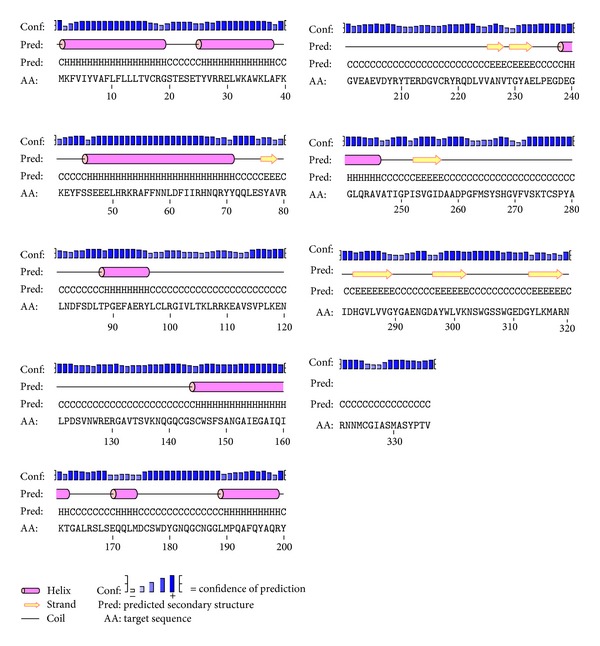
The predicted secondary structure of SeCP by using PSIPRED. There were 8 *α*-helixes, 7 *β*-strands, and 20 coils of the predicted secondary structure of SeCP.

PSIPRED v. 3.3 was used to predict the secondary structures of SeCP which had 8 *α*-helixes, 7 *β*-strands, and 20 coils (Figure 5).

### 3.5. Construction of 3D Model and Enzyme Activity Predicting

**Figure 6:**
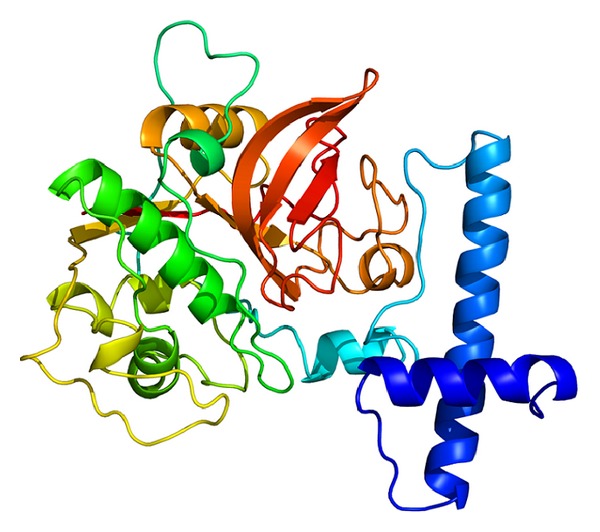
The 3D model of SeCP with highest confidence C-score, which estimates the quality of predicted models by I-TASSER.

Five models were set up for each protein by Dr. Zhang's lab [16]. We selected the model with the highest confidence C-score (Figure 6), which estimates the quality of predicted models by I-TASSER. It was calculated based on the significance of threading template alignments and the convergence parameters of the structure assembly simulations. C-score is typically in the range of [−5, 2], and a model with a C-score above 2 suggested a high confidence. Enzyme homologs in PDB predicted by I-TASSER showed that the most reliable enzyme classification (EC) number prediction was 3.4.22.43, with the highest Cscore^EC^ (0.664). Cscore^EC^ is the confidence score for the EC number prediction. Cscore^EC^ values range in between [0-1], where a higher score indicates a more reliable EC number prediction. The recombinant enzyme hydrolyzes proteins (serum albumin, collagen) and CA synthetic substrates (Z-Phe-Arg-NHMec > Z-Leu-Arg-NHMec > Z-Val-Arg-NHMec). The maximum activity of the enzyme was at pH 5.7 and was unstable at neutral pH. Compound E-64, leupeptin, and chicken cystatin are inhibitors of cysteine protease which belongs to peptidase family C1 (http://enzyme.expasy.org/EC/3.4.22.43).

### 3.6. Antigenic Epitope of SeCP

The sequence of SeCP was compared with the host's homologous sequences corresponding regional sequence by BepiPred 1.0b Server. The SeCP had 15 potential antigen epitopes (19aa–26aa, 44aa–50aa, 86aa–92aa, 118aa–128aa, 130aa–143aa, 151aa–155aa, 179aa–189aa, 199aa–205aa, 207aa–214aa, 231aa–243aa, 254aa–264aa, 276aa–280aa, 291aa–296aa, 305aa–312aa, and 332aa–336aa). Epitope prediction algorithm consensus was used to predict peptides that could stimulate human to induce effective and protective immune response against *S. erinaceieuropaei*, when the conserved HLA-restricted CD8+ T cells, epitopes of SeCP were predicted. The SeCP had 19 conserved peptides based on a high HLA allele binding score (percentile rank < 1) (Table 1).

### 3.7. Multiple Sequence Alignment and Molecular Evolution of SeCP

**Figure 7:**
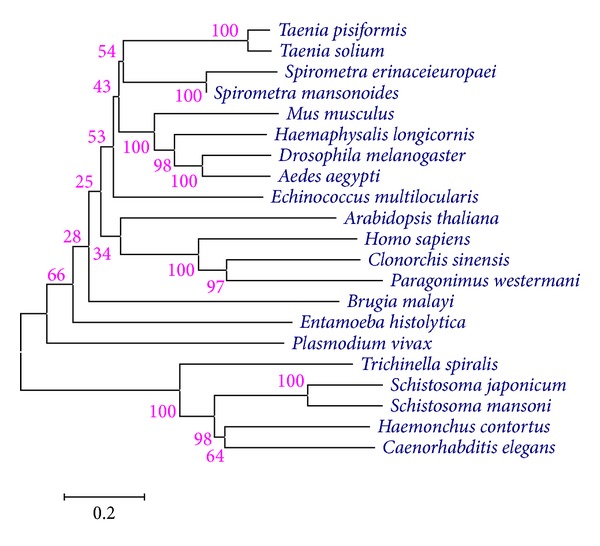
Neighbor-joining phylogenetic tree referred from cysteine protease amino acid sequence of *Spirometra erinacei*. Bootstrap values are indicated on branches.

Multiple sequence alignment and phylogenetic analysis of SeCP with the cysteine protease of other species were displayed in Figure 7. Based on the phylogenetic analysis of SeCP, *Spirometra erinaceieuropaei* has the closest evolutionary status with *Spirometra mansonoides*.

## 4. Discussion

Cysteine protease is a kind of proteolytic enzyme, which contains cysteine residues in the center of enzyme activity. It has been shown that the cysteine protease of many parasites acts extracellularly to help invade tissues and cells, to uptake nutrient, to hatch, or to evade the host immune system [24–26]. Cysteine protease is the key factor in the parasitic pathogenicity, either by inducing tissue damage and facilitating invasion or by empowering the parasites to salvage metabolites from host proteins [27, 28]. Cysteine protease has been detected in *S. erinacei* [29, 30]. The plerocercoids of *S. erinacei* is also known to secrete a large amount of cysteine proteases [31]. The cysteine protease from *S. erinacei* can hydrolyze collagen, hemoglobin, and immunoglobulin G (IgG) *in vitro* and may be concerned with digestion of host tissue in pathogenesis [32, 33]. Our previous study on 2DE analysis showed that the ES proteins of *S. erinacei* plerocercoids had a total of approximately 149 proteins spots with molecular weight varying from 20 to 115 kDa and isoelectric point (pI) from 3 to 7.5. When probed with sera from infected mice at 14 days after infection, seven protein spots with molecular weight of 23–31 kDa were recognized and analyzed by MALDI-TOF/TOF-MS. Three of seven spots were successfully identified and characterized as the same protein SeCP [13]. The SeCP might come from the excretory and secretory products and the cuticles (membrane proteins) and are directly exposed to the host's immune system and are the main target antiagens which induce the immune responses.

Based on the construction of full-length cDNA library of SeCP, the sequence of SeCP gene was of 1 053 bp length with a 1011 bp biggest ORF encoding 336-amino acid protein with a complete cathepsin propeptide inhibitor domain and a peptidase_C1A conserved domain. The predicted molecular weight and isoelectric point of the deduced SeCP protein were 37.87 Da and 6.47, respectively. Based on the phylogenetic analysis of SeCP, *Spirometra erinaceieuropaei* has the closest evolutionary status with *Spirometra mansonoides*. The secondary structure of SeCP contained has 8 *α*-helixes, 7 *β*-strands, and 20 coils. The SeCP had 15 potential antigenic epitopes and 19 HLA-I restricted epitopes. These predicted antigenic epitopes could provide important insights on the diagnostic antiagens and target molecular of antiparasitic drugs for sparganosis.

## Figures and Tables

**Table 1:** The predicted HLA restricted CD8^+^ T cell epitopes for SeCP.

Allele	Start	End	Peptide	Method	Percentile_rank
HLA-A*02:01	3	16	FVIYVAFLFLLLTV	Ann	0.3
HLA-A*02:01	263	272	FMSYSHGVFV	Consensus (ann/smm)	0.4
HLA-A*02:01	194	206	FQYAQRYGVEAEV	Ann	0.4
HLA-A*02:01	194	202	FQYAQRYGV	Consensus (ann/smm/comblib_sidney 2008)	0.5
HLA-A*02:01	188	197	GLMPQAFQYA	Consensus (ann/smm)	0.55
HLA-A*02:01	6	16	YVAFLFLLLTV	Consensus (ann/smm)	0.65
HLA-A*02:01	3	14	FVIYVAFLFLLL	Ann	0.8
HLA-A*02:01	9	16	FLFLLLTV	Consensus (ann/smm)	0.9
HLA-A*11:01	124	137	SVNWRERGAVTSVK	Ann	0.3
HLA-A*11:01	33	40	KAWKLAFK	Consensus (ann/smm)	0.45
HLA-A*11:01	307	316	SSWGEDGYLK	Consensus (ann/smm)	0.5
HLA-A*11:01	262	274	GFMSYSHGVFVSK	Ann	0.6
HLA-A*11:01	33	41	KAWKLAFKK	Consensus (ann/smm)	0.6
HLA-A*11:01	266	274	YSHGVFVSK	Consensus (ann/smm)	0.6
HLA-A*11:01	264	274	MSYSHGVFVSK	Consensus (ann/smm)	0.7
HLA-B*07:02	277	286	SPYAIDHGVL	Consensus (ann/smm)	0.35
HLA-B*07:02	317	330	MARNRNNMCGIASM	Ann	0.5
HLA-B*07:02	277	288	SPYAIDHGVLVV	Ann	0.6
HLA-B*07:02	106	117	KLRRKEAVSVPL	Ann	0.7
